# Reinforcement of Injectable Hydrogel for Meniscus Tissue Engineering by Using Cellulose Nanofiber from Cassava Pulp

**DOI:** 10.3390/polym15092092

**Published:** 2023-04-27

**Authors:** Rachasit Jeencham, Tulyapruek Tawonsawatruk, Piya-on Numpaisal, Yupaporn Ruksakulpiwat

**Affiliations:** 1 Research Center for Biocomposite Materials for Medical Industry and Agricultural and Food Industry, Nakhon Ratchasima 30000, Thailand; r_jeencham@hotmail.com; 2 Department of Orthopedics, Faculty of Medicine, Ramathibodi Hospital, Mahidol University, Bangkok 10400, Thailand; tulyapruek@gmail.com; 3 School of Orthopaedics, Institute of Medicine, Suranaree University of Technology, Nakhon Ratchasima 30000, Thailand; 4 School of Polymer Engineering, Institute of Engineering, Suranaree University of Technology, Nakhon Ratchasima 30000, Thailand

**Keywords:** cellulose nanofiber, PEO-PPO-PEO block copolymer-diacrylate, gelatin methacrylate, injectable hydrogel, meniscus tissue engineering

## Abstract

Injectable hydrogels can be applied to treat damaged meniscus in minimally invasive conditions. Generally, injectable hydrogels can be prepared from various polymers such as polycaprolactone (PCL) and poly (N-isopropylacrylamide) (PNIPAAm). Poly (ethylene oxide)-poly(propylene oxide)-poly(ethylene oxide) block copolymer-diacrylate (PEO-PPO-PEO-DA) is an interesting polymer due to its biodegradability and can be prepared as water-insoluble injectable hydrogel after curing with UV light at low intensity. However, mechanical and cell adhesion properties are not optimal for these hydrogels. For the improved mechanical performance of the injectable hydrogel, cellulose nanofiber (CNF) extracted from cassava pulp was used as a reinforcing filler in this study. In addition, gelatin methacrylate (GelMA), the denatured form of collagen was used to enhance cell adhesion. PEO-PPO-PEO-DA/CNF/GelMA injectable hydrogels were prepared with 2-hydroxy-1-(4-(hydroxy ethoxy) phenyl)-2-methyl-1-propanone as a photoinitiator and then cured with UV light, 365 nm at 6 mW/cm^2^. Physicochemical characteristics of the hydrogels and hydrogels with CNF were studied in detail including morphology characterization, pore size diameter, porosity, mechanical properties, water uptake, and swelling. In addition, cell viability was also studied. CNF-reinforced injectable hydrogels were successfully prepared after curing with UV light within 10 min with a thickness of 2 mm. CNF significantly improved the mechanical characteristics of injectable hydrogels. The incorporation of GelMA into the injectable hydrogels improved the viability of human cartilage stem/progenitor cells. At optimum formulation, 12%PEO-PPO-PEO-DA/0.5%CNF/3%GelMA injectable hydrogels significantly promoted cell viability (>80%) and also showed good physicochemical properties, which met tissue engineering requirements. In summary, this work shows that these novel injectable hydrogels have the potential for meniscus tissue engineering.

## 1. Introduction

Scaffolds for tissue engineering act as structural support to repair damaged tissues by providing cell adhesion and proliferation, and subsequent development of new tissue. Currently, marketed scaffolds for tissue engineering are in various forms such as sponge, nanofibrous, 3D printing, and solid hydrogel scaffolds. They are typically produced in porous solid templates before implantation into the target tissue. The benefit of solid scaffold forming before implantation is that it is a simple and not complicated process. However, the implantation into the target tissue is a difficult and complicated procedure. This is because the insertion and implantation of these solid templates need an open incision that requires a long period of time to heal. To overcome the limitations of solid scaffold forming before implantation, it is important to develop a novel scaffold for reducing the surgical wound area and healing time by minimally invasive surgery instead of open incisions. Recently, fibrin glue has been applied as an injectable fibrin scaffold. The fibrin glue is composed of fibrinogen and thrombin obtained from human and animal blood. The fibrinogen and thrombin solutions were in separate syringes and then passed through a small incision and together injected into the target tissue. The combination of the two solutions will become a solid gel within a few minutes. The benefit of injectable fibrin scaffold is good biocompatibility, cell adhesion, and proliferation [1]. However, most of them fail to achieve to be used because of a risk of immune response and poor mechanical properties [1]. To overcome the limitations of fibrin glue and to reduce the usage of products from human and animal blood, we have proposed to develop a novel artificial fibrin glue from biodegradable materials.

**Figure 1:**
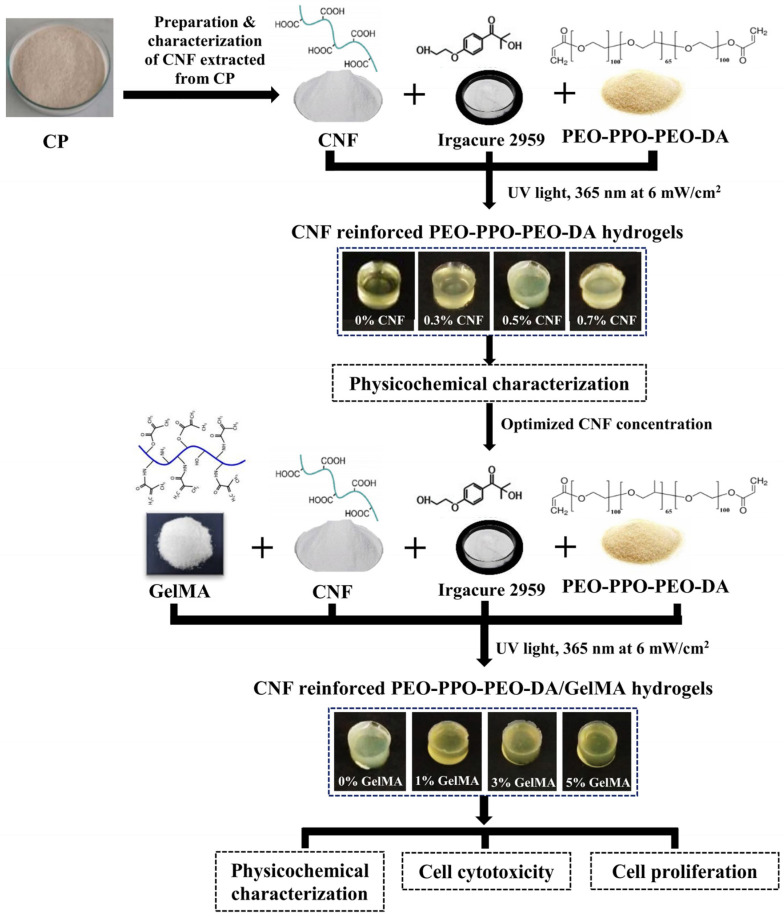
Overview of the study design.

Poly (ethylene oxide)-poly(propylene oxide)-poly(ethylene oxide) block copolymer-diacrylate (PEO-PPO-PEO-DA) is one of the interesting biodegradable synthetic polymers which is water insoluble hydrogel after curing with UV light at low intensity [2]. However, this injectable hydrogel has only slightly higher mechanical properties than fibrin glue. Moreover, it showed poor cell adhesion and proliferation [3]. To improve the properties of this hydrogel, reinforcement agents and enhancing cell adhesion materials are needed to incorporate. In this study, we are interested in using cellulose nanofiber (CNF) from cassava pulp (CP) extraction as a reinforcing agent because of its unique mechanical properties, biodegradability, and non-toxicity [4]. In previous studies, many researchers confirmed that CNF enhanced the mechanical properties of various polymers hydrogels, such as gelatin, chitosan, and sodium alginate for tissue engineering. Qingxiu, L. et al. (2020) [5] found that 0.80% (*w*/*v*) CNF significantly improved the compression strength of 10% (*w*/*v*) gelatin hydrogel. Doench, I. et al. (2019) [6] investigated and found that Young’s modulus of the 2% (*w*/*v*) chitosan hydrogel was significantly increased when reinforced with CNF. It was also found that when increasing the cellulose nanofiber concentration from 0.2% (*w*/*v*) to 0.4% (*w*/*v*), Young’s modulus of the chitosan hydrogel significantly increased. Olav, A. et al. (2017) [7] studied the mechanical properties of CNF-reinforced sodium alginate hydrogels. The compression strength of the hydrogel increased with incorporating CNF from 0.15 to 0.75% (*w*/*v*). In addition, previous research evidenced that CNF was not cytotoxic in cells. Kim, H.J. et al. (2018) [8] found that CNF hydrogel was non-cytotoxic in pre-osteoblasts (MC3T3-E1) cells. CP has long been known as the natural source of CNF production. It is obtained as the residual material of cassava plant, which is one of the major harvests with the annual production; however, its wasted amount is relatively high, and this can lead to environmental problems. There are three reasons why we decided to choose cassava as reinforcement material. Firstly, 28 million tons of cassava were produced in 2022, which was significant economically, especially in developing countries [9]. Secondly, the content of cellulose in CP can be as high as 20%, and thus, it is appropriate to extract CNF from CP [10]. Finally, there is a huge amount of CP wasted, around 7.3 million tons/year, and CP became a rich source for the production of CNF without shortage [11]. Therefore, we supposed CP is a good material to apply CNF into applications in medical industries instead of leaving most of it to be used as animal feed and wasted in wet weather just because it is cheap. In addition, gelatin methacrylate (GelMA) can be considered as a model of the extracellular matrix (ECM). GelMA is prepared by covalent binding gelatin and methacrylic anhydride (MA). Gelatin is similar to collagen (a main component of the ECM). Following collagen isolation, gelatin can be obtained by alkaline or acid hydrolysis. It is important to keep in mind that, following the denaturalization step, gelatin turns into a linear structure, made out of Gly-X-Y (mainly proline and hydroxyproline) sequences. The arginylglycylaspartic acid (RGD) motif, responsible for cell adhesion to ECM and cell proliferation is also present in the gelatin [12]. In addition, many studies reported that GelMA represents one of the most promising candidates for the development of scaffold material for tissue repair owing to its various advantages: water-insoluble, enhancement of cell adhesion and proliferation, biodegradability, non-toxicity, and promising in vitro and in vivo tissue regeneration [13]. Celikkin, N. et al. (2017) [14] studied the effect of GelMA concentration (5 and 10% *w*/*v*) on osteogenic differentiation in rat mesenchymal stem cells (MSCs). They discussed that MSCs in both 5% (*w*/*v*) GelMA and 10% (*w*/*v*) GelMA hydrogels showed good osteogenic differentiation. Especially, 5% (*w*/*v*) GelMA hydrogel encouraged higher MSC attachment. Nowadays, there are very few reports on the development of injectable hydrogels for meniscus tissue engineering [15,16,17], especially; there are no reports on CNF-reinforced PEO-PPO-PEO-DA/GelMA injectable hydrogel for meniscus tissue engineering. In this work, we have developed CNF-reinforced PEO-PPO-PEO-DA/GelMA injectable hydrogels for meniscus tissue engineering. The effect of CNF and GelMA on the physicochemical properties of a hydrogel, such as morphology, pore size diameter, porosity, mechanical properties, water uptake and swelling, gel fraction, and chemical interaction were investigated. In addition, cell cytotoxicity and proliferation were also studied. The overview of the study design is shown in Figure 1.

## 2. Materials and Methods

### 2.1. Materials

Poly (ethylene oxide)-poly(propylene oxide)-poly(ethylene oxide) block copoly-mer-diacrylate (PEO-PPO-PEO-DA, MW 12,500 g/mol), gelatin methacrylate (GelMA, Type A: porcine skin, gel strength 300 g Bloom, degree of substitution 60%), Fibrinogen (plasminogen-depleted human plasma), thrombin (bovine plasma) and 2-Hydroxy-4′-(2-hydroxyethoxy)-2-methylpropiophenone (Irgacure 2959) MTT (3-[4,5-dimethylthiazol-2-yl]-2,5 diphenyl tetrazolium bromide), Dulbecco’s modified Eagle’s medium (DMEM)-high glucose, PLTGold^®^ human platelet lysate, and L-glutamax were purchased from Sigma-Aldrich Corporation (St. Louis, MI, USA). Cassava pulp was supplied by Sanguan Wongse Industries Co., Ltd. (Nakhon Ratchasima, Thailand). Sodium hydroxide (NaOH, 98%) was purchased from AGC Chemicals (Thailand) Co., Ltd. (Bangkok, Thailand). Sodium chlorite (NaClO_2_, technical grade, 80%) was purchased from Thermo Fisher Scientific Inc. (Waltham, MA, USA). Calcium chloride (CaCl_2_) and glacial acetic acid were obtained from RCI Labscan Co., Ltd. (Bangkok, Thailand).

### 2.2. Preparation of CNF

Cassava pulp (CP) was ground and sieved with a mesh size of 150–250 µm. Then, an oven was used to dry CP at 120 °C for 24 h to remove excess moisture. After that, 200 g of CP was soaked in 4 L of 4 wt% sodium hydroxide (NaOH) solution at 80 °C for 2 h under mechanical stirring and then washed with deionized water several times until reaching a pH of 7. Finally, the CP after alkali treatment was dried for 1 day in an oven at 80 °C. Alkali-treated cassava pulp (TCP) was produced following alkali treatment. After alkali treatment, the TCP was mixed with a solution of acetate buffer (27 g NaOH and 75 mL glacial acetic acid, diluted to 1 L of distilled water) and aqueous chlorite (1.7% (*w*/*v*) NaClO_2_ in water) in equal amounts to conduct the bleaching treatment. The ratio of TCP and bleaching solution was 1:20. Bleaching was performed at 80 °C for 6 h. Following bleaching, TCP was washed repeatedly in deionized water until the pH of the bleached TCP became neutral. The bleached TCP was put into an oven to dry at 80 °C for 24 h and then grinding and sieving processes were conducted with a mesh size of 38–63 µm. The sample resulting from the bleaching treatment was called bleached cassava pulp (BCP). CNF was prepared by an application of the high-pressure homogenizing process. Briefly, 0.5% (*w*/*v*) BCP aqueous dispersion was dispersed in phosphate-buffered saline (PBS) solution. The suspension was treated with a high-pressure homogenizer (Microfluidics M-110EH-30, Microfluidics International Corporation, Westwood, MA, USA) to separate the fibril bundles from BCP. The homogenization pressure was adjusted to 25,000 psi, and the homogenization number of cycles was 15 cycles.

### 2.3. Preparation of CNF-Reinforced PEO-PPO-PEO-DA/GelMA Injectable Hydrogels

To find the optimum CNF concentration for reinforcing in PEO-PPO-PEO-DA hydrogels, the physicochemical properties of injectable hydrogels were investigated using varying CNF concentrations. The hydrogels were prepared by solution mixing. In the first step, 0.3% (*w*/*v*) IR2959 as a photoinitiator was added into CNF suspension at 60 °C under stirring of 500 rpm, for 30 min. The CNF was added with the following concentrations: 0, 0.3, 0.5, and 0.7% (*w*/*v*). 12% (*w*/*v*) PEO-PPO-PEO-DA was dissolved in the CNF suspension at 4 °C for 24 h. After that, the liquid mixtures were stirred for 30 min, 500 rpm at room temperature. The liquid mixtures were injected into the mold and cured with UV light, 365 nm at 6 mW/cm^2^ for 10 min, after curing the physicochemical properties of hydrogels were characterized. 

After that, the optimum CNF concentrations were selected to prepare CNF/PEO-PPO-PEO-DA/GelMA injectable hydrogels. In the first step, two times of using IR2959 or 0.6% (*w*/*v*) IR2959 as a photo initiator was added into CNF suspension at 60 °C under stirring of 500 rpm for 30 min. Two times using PEO-PPO-PEO-DA or 24% (*w*/*v*) PEO-PPO-PEO-DA was dissolved in the CNF/IR2959 suspension at 4 °C for 24 h. GelMA with various as 2, 6, and 10% (*w*/*v*) was dissolved in the CNF/IR2959 suspension at 60 °C for 1 h. The prepared PEO-PPO-PEO-DA in CNF/IR2959 suspension and prepared GelMA in CNF/IR2959 suspension were mixed for 30 min, 500 rpm at room temperature. Finally, the mixtures gave IR2959 of 0.3% (*w*/*v*), PEO-PPO-PEO-DA concentration of 12% (*w*/*v*), and GelMA concentration of 1, 3, and 5% (*w*/*v*), respectively. The liquid mixtures were injected into the mold and cured with UV light, 365 nm at 6 mW/cm^2^ for 10 min. After curing, the physicochemical properties of injectable hydrogels and cell viability assessment were characterized.

### 2.4. Preparation of Fibrin Glue

In this study, CNF-reinforced PEO-PPO-PEO-DA injectable hydrogels were compared with the injectable fibrin glue. The fibrin glue was prepared according to Numpaisal et al. (2016) [18]. Briefly, fibrinogen, 20 mg/mL was dissolved in PBS. Thrombin and CaCl_2_, with final concentration values of 5.3 U/mL and 4.12 mM, respectively, were added for initiating polymerization to an end fibrinogen concentration of 10 mg/mL. Both fibrinogen and thrombin/CaCl_2_ solutions were injected into the mold, and the fibrin glue was allowed for polymerization at 37 °C.

### 2.5. Characterization of Cellulose Nanofiber

#### 2.5.1. Morphology, Diameter, and Length

A field emission scanning electron microscope (FE-SEM) (Carl Zeiss AURIGA^®^, Thuringia, Germany) was employed for the morphology and diameter of CNF. Samples were sputter-coated with carbon on the surface. Images were acquired with a voltage of 5 kV. The CNF size was determined by ImageJ software (Wayne Rasband NIH, Washington, DC, USA). FE-TEM (Talos™ F200X, Thermo Fisher Scientific Inc., Waltham, MA, USA) was applied to determine the length of CNF. A drop of the diluted suspension was cast onto the surface of a clean copper grid and covered with a thin carbon film. TEM images of the CNF were negatively stained in Uranyless for 1 min, then washed in distilled water. Drying the sample at normal temperature was vital before FE-TEM analysis. The length of CNF was also measured by ImageJ software in 100 random fibers, and the diameter distribution curves of CNF were plotted by OriginLab software, (OriginPro^®^ 2021b, OriginLab Corporation, Northampton, MA, USA).

#### 2.5.2. Structure

The structural and chemical composition of CP, BCP, and CNF were characterized by a Tensor 27 FTIR spectrometer (Bruker Optics, Ettlingen, Germany). FTIR was performed in the spectra range of 4000–400 cm^−1^.

#### 2.5.3. Crystallinity

X-ray diffraction was used for crystallinity assessment of CP, BCP, and CNF’s determination. Each sample in the form of milled powder was placed on the sample holder and flattened for uniform X-ray exposure. Sample analysis was carried out by an X-ray diffractometer (D8-Advance Bruker AXS GmbH, Ettlingen, Germany) at RT with a monochromatic CuK radiation source (λ = 0.1539 nm) in the step-scan mode with a 0.2° angle ranging from 5° to 40° with a step of 0.02 and scanning time of 0.5 min. The Segal method was applied to obtain the crystallinity index (CrI) of the [19], as shown in Equation (1).

(1) $$\mathrm{CrI}\;(\%)=\frac{(I_{200}\;-\;I_{am})}{I_{200}\;}\;\times \;100$$

*I_200_* the maximum intensity of the (*2 0 0*) reflection at 2θ of 22°–23°, and *I_am_* is the lowest intensity of diffraction at 2θ of 18°–19° for the amorphous part.

### 2.6. Physicochemical Characterization of Injectable Hydrogel

#### 2.6.1. Morphology and Pore Size Diameter

The morphology and pore size of the top surface and the cross-section of the hydrogels were assessed by FE-SEM, 5 kV voltage, (Carl Zeiss AURIGA^®^, Thuringia, Germany), after gold sputtering. The pore size diameter of samples was determined by ImageJ software (Wayne Rasband NIH, Washington, DC, USA) in 100 random pores, and the pore size diameter distribution diagram of hydrogel samples was plotted by OriginLab software (OriginPro^®^ 2021b, OriginLab Corporation, Northampton, MA, USA).

#### 2.6.2. Porosity

Hydrogel porosity was assessed by means of the liquid displacement method. Briefly, the lyophilized hydrogel was soaked in a defined volume (V_1_) of ethanol in a 5 mL cylinder for 10 min. The total volume of ethanol and sample was recorded as V_2_. The ethanol-impregnated sample was removed from the cylinder, and the residual volume of ethanol was recorded as V_3_. The porosity was calculated according to Equation (2). The sample number was three per group (*n* = 3).

(2) $$\mathrm{Porosity}\;(\%)=\frac{V1\;-V3}{V2\;-V3}\;\times \;100$$

#### 2.6.3. Gel Fraction

Hydrogels were dried in an oven at 40 °C for one day and then weighed (dry weight before immersion). After drying, the hydrogels were hydrated in distilled water at 37 °C for 24 h to remove the soluble fraction and dried at 40 °C for 24 h (dry weight after immersion), then the gel fraction of hydrogel samples was calculated as shown in Equation (3). Experiments were carried out in triplicate (*n* = 3).

(3) $$\mathrm{Gel}\;\mathrm{fraction}\;(\%)=\frac{\mathrm{dry}\;\mathrm{weight}\;\mathrm{before}\;\mathrm{immersion}}{\mathrm{dry}\;\mathrm{weight}\;\mathrm{after}\;\mathrm{immersion}}\;\times \;100$$

#### 2.6.4. Water Uptake and Swelling

The hydrogels were left soaking in distilled water at 37 °C for one day and then weighed (wet weight). The wet hydrogels were then dried in an oven at 40 °C for 24 h (dry weight after immersion). The water uptake and swelling of the hydrogels were obtained according to the following Equations (4) and (5), respectively. All experiments were performed in triplicate (*n* = 3).

(4) $$\mathrm{Water}\;\mathrm{uptake}\;(\%)=\frac{\left(\mathrm{wet}\;\mathrm{weight}\;-\;\mathrm{dry}\;\mathrm{weight}\;\mathrm{after}\;\mathrm{immersion}\right)}{\mathrm{wet}\;\mathrm{weight}}\;\times \;100$$

(5) $$\mathrm{Water}\;\mathrm{swelling}\;(\%)=\frac{\left(\mathrm{wet}\;\mathrm{weight}\;-\;\mathrm{dry}\;\mathrm{weight}\;\mathrm{after}\;\mathrm{immersion}\right)}{\mathrm{dry}\;\mathrm{weight}\;\mathrm{after}\;\mathrm{immersion}}\;\times \;100$$

#### 2.6.5. Mechanical Properties

The compressive strength of the hydrogels (diameter = 5 mm and height = 4 mm) was evaluated with a texture analyzer (TA.XT-PLUS, London, UK) with a load cell of 5 kg, a compression speed of 3 mm/min and pressure distance 80% strain. The ultimate tensile strength and elongation at break of the hydrogel were also determined using a texture analyzer (TA.XT-PLUS, Texture Technologies Corp., London, UK) with a load cell of 5 kg. The samples (length = 30 mm, width = 5 mm, and thickness = 1.5 mm) were attached to the central part of the tensile grip jaw. The upper sample (10 mm) and the lower sample (10 mm) were gripped by the tensile grip jaw and finally, a gauge length for tensile testing was 10 mm. The tensile tests were performed at a speed rate of 10 mm/min. All the tests represented the average results from three test specimens (*n* = 3).

#### 2.6.6. Chemical Composition and Interactions

FTIR measurements were conducted on the hydrogels in a range from 4000 to 400 cm^−1^ with a Tensor 27 FTIR spectrometer (Bruker Optics, Ettlingen, Germany).

### 2.7. In Vitro Cell Culture Studies

#### 2.7.1. Cell Cytotoxicity

The extract dilution method is more commonly adopted for the in vitro cytotoxicity evaluation of materials and devices used in the body since it can be applied to a wide variety of raw materials and finished products that may release toxins from exposed surfaces (un-crosslinked polymer solutions and residual crosslinker). The liquid samples were autoclaved at 121 °C, 15 psi pressure for 20 min. The liquid samples were pipetted into a 96-well plate and then induced into the hydrogels by UV irradiation, 365 nm at 6 mW/cm^2^ for 10 min in a laminar flow cabinet. Cytotoxicity was determined by human cartilage stem/progenitor cell (CSPCs) viability after incubation in an extracted medium of the hydrogels for 24 h. The cell viability was assessed with an MTT (3-[4,5-dimethylthiazol-2-yl]-2,5 diphenyl tetrazolium bromide) assay. The extracted medium of the hydrogels was derived from hydrogel immersion in cell culture medium (Dulbecco’s modified Eagle’s medium (DMEM)-high glucose, 5% PLTGold^®^ Human platelet lysate, 1% L-glutamax and 1% antibiotic) for 24 h. The CSPCs were seeded onto 96-well plates at 2.5 × 10^4^ cells per well in 200 µL of a culture medium and incubated at 37 °C, 5% CO_2_ for 24 h. After 24 h of cell incubation, the old culture medium was removed and 200 µL of the extracted medium of the hydrogels was then added to the 96-well plates. The cells seeded 96-well plates were incubated in an extracted medium of the hydrogels for up to 24 h at 37 °C, 5% CO_2_. Then, the cells were cleaned with PBS two times, and 100 µL of MTT solution (5 mg MTT/mL of medium) was added. After a 2 h reaction time, MTT formazan was extracted with dimethyl sulfoxide (DMSO) for 10 min, followed by measuring the absorbance of the extracted medium at 570 nm with a microplate reader (Infinite M200Pro, Tecan Group Ltd., Männedorf, Switzerland). All the results were expressed as relative viability compared to cells grown in the culture medium without hydrogel immersion (control). The cell viability percentage was calculated by dividing MTT formazan absorbance for each sample by the absorbance formazan for control and multiplying this number per 100 as in Equation (6). Assays were carried out in triplicate for each sample (*n* = 3).

(6) $$\;\mathrm{Cell}\;\mathrm{viability}\;(\%)=\frac{\mathrm{Absorbance}\;\mathrm{of}\;\mathrm{sample}}{\mathrm{Absorbance}\;\mathrm{of}\;\mathrm{control}}\;\times \;100$$

#### 2.7.2. Cell Proliferation

Cell proliferation in hydrogels was determined by CSPCs viability at days 1, 7, and 14. The MTT assay was used for cell viability studies. Samples were autoclaved at 121 °C, 15 psi pressure for 20 min, and pipetted into a 96-well plate and then induced into the hydrogels by UV irradiation, 365 nm at 6 mW/cm^2^ for 10 min in a laminar flow cabinet. The CSPCs were seeded onto the hydrogels in 96-well plates at 2.5 × 10^4^ cells per well in 200 µL of cell culture medium. Culture of cell seeded hydrogels was performed in DMEM-high glucose, 5% PLTGold^®^ human platelet lysate, 1% L-glutamax, and 1% antibiotic, put in 37 °C with a 5% CO_2_ humidified incubator. After the cell culture in the hydrogels for 1, 7, and 14 days, the cells were washed with PBS twice, and 100 µL of MTT solution (5 mg MTT/mL of medium) was added. MTT formazan was extracted 2 h later with dimethyl sulfoxide (DMSO) for 10 min, followed by measuring absorbance of the extracted solution at 570 nm with a microplate reader (Infinite M200Pro, Tecan Group Ltd., Männedorf, Switzerland). All the results were expressed as relative viability compared to cells grown in the culture medium without hydrogel immersion (control). The cell viability percentage was calculated by dividing MTT formazan absorbance for each sample by the absorbance formazan for control and multiplying this number per 100 as in Equation (6). Assays were performed in triplicate for each sample (*n* = 3).

### 2.8. Statistical Analysis

The results were expressed as mean ± standard deviation. All quantitative data were analyzed with one-way ANOVA and paired *t*-test followed by Tukey’s post hoc comparison test. To identify statistical differences between the comparison groups, a *p*-value < 0.05 was considered statistically significant.

## 3. Results and Discussion

### 3.1. Characterization of Cellulose Nanofiber

**Figure 2:**
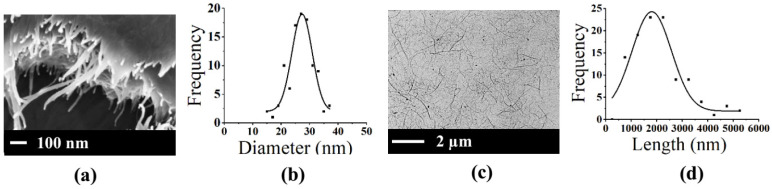
(**a**) FE-SEM image of CNF, (**b**) Diameter distribution of CNF, (**c**) FE-TEM image of CNF, and (**d**) Length distribution of CNF with 25,000 psi 15 cycles.

Figure 2a,c display the FE-SEM and FE-TEM micrographs of CNF, respectively. Both figures show that the appearance of CNF was obtained from the homogenizing technique in fiber form. The diameter and length of CNF obtained from FE-SEM and FE-TEM images, respectively, were measured by ImageJ software. From the diameter and length distribution shown in Figure 2b and Figure 2d, respectively, the CNF with homogenizing technique at 25,000 psi and 15 cycles reveal an average diameter of 27 nm, diameters in the range of 15–37 nm, the average length of 1802 nm and length in the range of 507–5123 nm.

**Figure 3:**
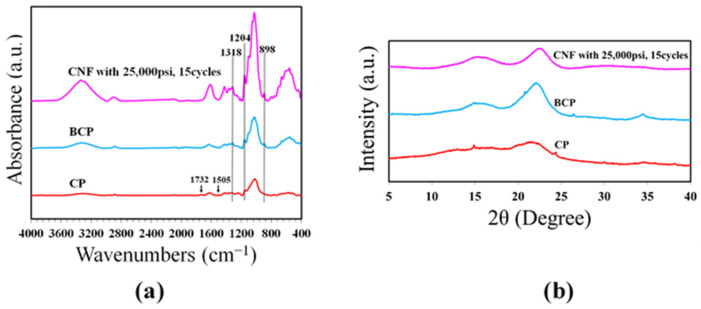
(**a**) FTIR spectra of CP, BCP, and CNF with 25,000 psi 15 cycles and (**b**) XRD of CP, BCP, and CNF with 25,000 psi 15 cycles.

Figure 3a shows the FTIR spectra of CP, BCP, and CNF with homogenizing technique. The peaks between 3600–3000 cm^−1^ of all samples corresponded to –OH stretching. This band was related to intermolecular hydrogen bonds of cellulose [15,16]. The peaks at 1318 cm^−1^, 1204 cm^−1^, and 898 cm^−1^ of BCP and CNF were attributed to CH_2_ wag, OH-deformation, and beta glycosidic linkages of the glucose ring; these bands were referred to the structure of the cellulose components [20,21]. The peaks at 1732 cm^−1^ and 1505 cm^−1^ of raw cassava pulp corresponded to C=O stretching and C=C aromatic ring; these referred to lignin and hemicellulose [20,21]. Apparently, these peaks disappeared in the pretreated cassava pulp and CNF, indicating partial removal of hemicelluloses and lignin from the structure of fibers leading to higher purity of cellulose. Furthermore, no significant differences were observed among the spectra of BCP and CNF. The results proved that the cellulose molecular structure remained unchanged with the homogenizing method.

From the XRD curve shown in Figure 3b, the crystalline peaks of all samples showed 2θ at 16° and 22°. The crystalline peak of the CP had the lowest intensity. From the XRD pattern, the crystallinity index (CI) of all samples could be determined. The Segal method was used to calculate the CI with the principal diffraction peak of cellulose I around 22° and the lowest intensity at around 18°. The CP showed the lowest CI of 19%, since it contains a great amount of amorphous area. On the other hand, the BCP and CNF give high crystallinity of 43–46% (Table 1). This occurred due to the deletion of hemicelluloses and lignin bound to the fibers of cellulose.

### 3.2. Effect of CNF Concentration on Physicochemical Properties of PEO-PPO-PEO-DA Injectable Hydrogels

**Figure 4:**
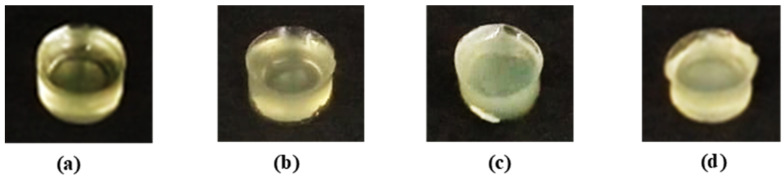
Appearances of injectable hydrogels (**a**) 12% PEO-PPO-PEO-DA, (**b**) 12%PEO-PPO-PEO-DA/0.3%CNF, (**c**) 12%PEO-PPO-PEO-DA/0.5%CNF, and (**d**) 12%PEO-PPO-PEO-DA/0.7%CNF.

**Figure 5:**
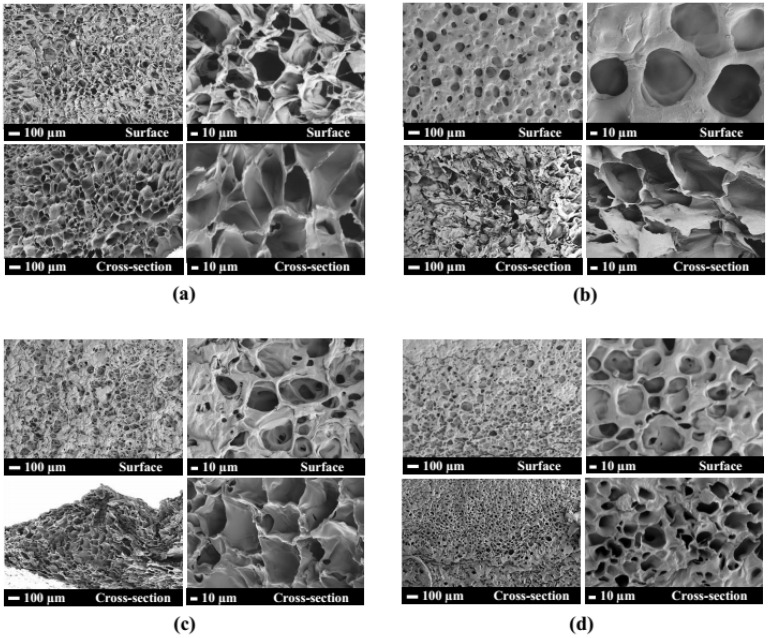
FE-SEM micrographs of surface and cross-section of (**a**) 12%PEO-PPO-PEO-DA, (**b**) 12%PEO-PPO-PEO-DA/0.3%CNF, (**c**) 12%PEO-PPO-PEO-DA/0.5%CNF, and (**d**) 12%PEO-PPO-PEO-DA/0.3%CNF injectable hydrogel.

**Figure 6:**
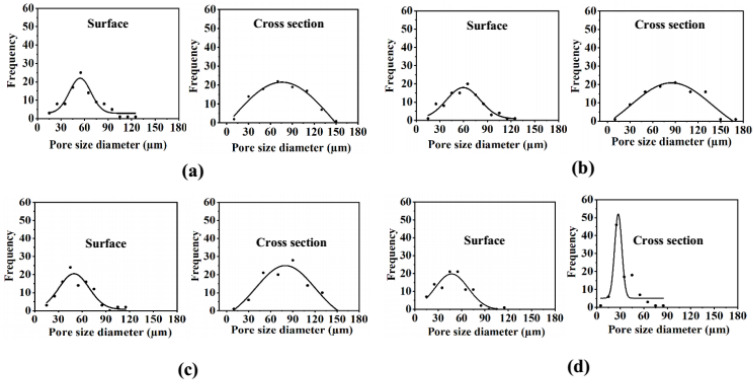
Pore size diameter distribution of the surface and cross-section of injectable hydrogel of (**a**) 12%PEO-PPO-PEO-DA, (**b**) 12%PEO-PPO-PEO-DA/0.3%CNF, (**c**) 12%PEO-PPO-PEO-DA/0.5%CNF, and (**d**) 12%PEO-PPO-PEO-DA/0.3%CNF injectable hydrogel.

PEO-PPO-PEO-DA injectable hydrogels with several CNF concentrations at 0, 0.3, 0.5, and 0.7% (*w*/*v*) were successfully prepared after curing with UV light within 10 min. All the injectable hydrogels were uniformly translucent, non-brittle, and strong enough to handle without deformation (Figure 4). The morphologies of hydrogels with different CNF concentrations were characterized by FE-SEM. The FE-SEM images shown in Figure 5 reveal the porous structures of the hydrogels, which play a fundamental role in cell proliferation. The FE-SEM image of the surface and cross-section of all the hydrogels show highly microporous structures with an interconnecting network, pore size diameter of a surface range of 11–128 µm and a cross-section range of 9–168 µm (Figure 6 and Table 2). All the hydrogels show a porosity of 65–93% (Table 2). As a result of augmenting the CNF concentration, porosity in the hydrogels diminishes, and the hydrogel structure becomes tighter, suggesting that the CNF may be dispersed in the polymeric structure. Lately, scaffolds displaying pores from 5 to 350 µm in diameter and porosity of ≥ 55% have been used for tissue regeneration [22]. This suggests that all the hydrogels show an adequate porosity and pore siz e suitable for chondrocyte growth and proliferation.

**Figure 7:**
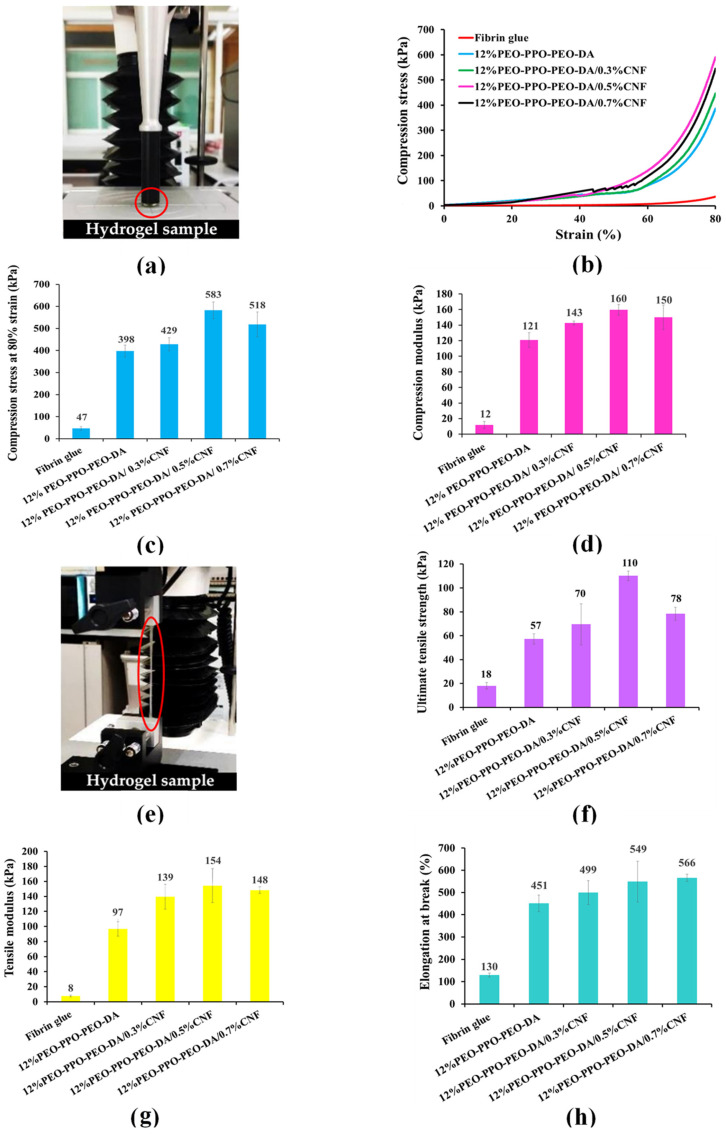
(**a**) Compression characterization, (**b**) Compression stress–strain curve, (**c**) Compression stress at 80% strain, (**d**) Compression modulus, (**e**) Tensile characterization, (**f**) Ultimate tensile strength, (**g**) Tensile modulus and (**h**) Elongation at break of PEO-PPO-PEO-DA, PEO-PPO-PEO-DA/CNF injectable hydrogels and fibrin glue. The red circle indicates hydrogel sample.

The mechanical properties of injectable hydrogels must be taken into consideration in relation to resistance to damage when scaffolds are applied for tissue engineering. In this study, the CNF-reinforced PEO-PPO-PEO-DA injectable hydrogels were compared with the conventional injectable hydrogel, fibrin glue. Figure 7 shows the poor mechanical properties of the fibrin glue: compression strength of 47 kPa, compression modulus of 12 kPa, ultimate tensile strength of 18 kPa, tensile modulus of 8 kPa, and elongation at break of 130%. Especially, all the CNF- reinforced PEO-PPO-PEO-DA injectable hydrogels show remarkably higher mechanical properties than those of the fibrin glue. Interestingly, the mechanical properties of the injectable hydrogels significantly improved with the increase of CNF from 0 to 0.5% (*w*/*v*). However, the mechanical properties of the hydrogels were slightly reduced with the increase of CNF from 0.5 to 0.7% (*w*/*v*). All the CNF-reinforced PEO-PPO-PEO-DA injectable hydrogels show good mechanical properties: compression strength (429–583 kPa), compression modulus (143–160 kPa), ultimate tensile strength (70–110 kPa), tensile modulus (139–154 kPa) and elongation at break (499–566%) which met the hydrogel scaffold requirements. In addition, the CNF-reinforced PEO-PPO-PEO-DA injectable hydrogels exhibit remarkably better mechanical properties than other hydrogel materials (summarized in Table 3), which confirms the improvements brought by incorporating CNF in the semi-interpenetrating network. Subsequently, the CNF-reinforced PEO-PPO-PEO-DA injectable hydrogels were selected for subsequent experiments.

Water uptake and swelling are fundamental for cell growth on hydrogels aimed at biological applications. Typically, water uptake and swelling of hydrogels for use as tissue engineering should be more than 50% and 100%, respectively [29]. As shown in Table 4, all hydrogels show high water uptake of 86–87%. However, after augmenting CNF concentration, the water uptake capacity of the hydrogels slightly decreased because of additional physical interaction due to hydrogen bonds and the resulting decrease in porosity [29]. Table 4 shows the water swelling of PEO-PPO-PEO-DA and CNF-reinforced PEO-PPO-PEO-DA injectable hydrogels. All of the hydrogels show high water swelling of 618–643% hinting high porosity for the hydrogels. However, the swelling characteristics of PEO-PPO-PEO-DA hydrogel were higher than for other hydrogels. Again, the reason may be found in the CNF creating a tighter, denser network and diminishing porous content [29].

Gel fraction is the degree of crosslinking of hydrogels produced by UV irradiation. Typically, gel fraction of hydrogels for use as tissue engineering should be more than 80% [30]. All the hydrogels show high gel fraction of >80% (Table 4), which meet the requirement, indicating that the hydrogels are cross-linked three-dimensional hydrophilic polymer networks capable of swelling without losing integrity when in an aqueous environment.

As evidenced by all the results, this indicates that 0.5% (*w*/*v*) CNF-reinforced polymer matrix gives the highest mechanical properties without deterioration of other properties that meet the physicochemical properties of tissue engineering requirements. Therefore, CNF at 0.5% (*w*/*v*) was selected for reinforcing in PEO-PPO-PEO-DA/GelMA injectable hydrogels.

### 3.3. Characterization of CNF-Reinforced PEO-PPO-PEO-DA/GelMA Injectable Hydrogels

The purpose of incorporating GelMA into PEO-PPO-PEO-DA/CNF injectable hydrogel was to promote cell adhesion and proliferation. In this study, the effects of GelMA concentration on physicochemical properties were investigated. In addition, cell cytotoxicity, and cell proliferation were also studied.

#### 3.3.1. Physicochemical Characterization

##### Appearance

**Figure 8:**
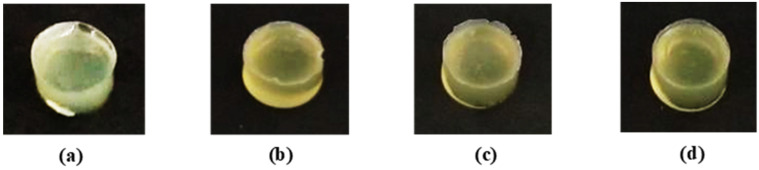
Appearances of (**a**) 12%PEO-PPO-PEO-DA/0.5%CNF, (**b**) 12%PEO-PPO-PEO-DA/0.5%CNF/1%GelMA, (**c**) 12%PEO-PPO-PEO-DA/0.5%CNF/3%GelMA, and (**d**) 12%PEO-PPO-PEO-DA/0.5%CNF/5%GelMA injectable hydrogels.

PEO-PPO-PEO-DA/CNF injectable hydrogels with various GelMA concentrations at 0, 1, 3, and 5% (*w*/*v*) were successfully prepared after curing with UV light within 10 min. All the injectable hydrogels were uniformly translucent, non-brittle, and with enough strength to be handled without showing deformation (Figure 8).

##### Morphology, Pore Size Diameter, and Porosity

**Figure 9:**
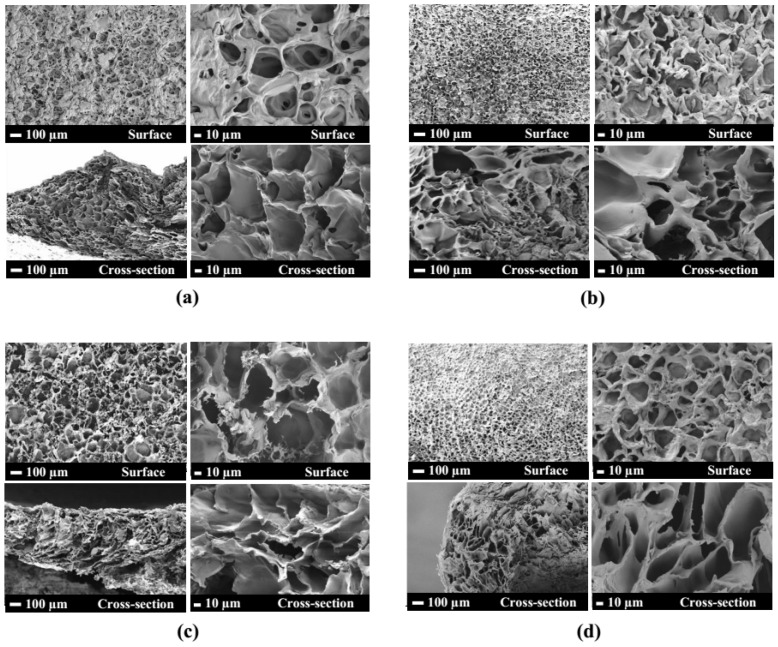
FE-SEM micrographs of the surface and cross-section of (**a**) 12%PEO-PPO-PEO-DA/0.5%CNF, (**b**) 12%PEO-PPO-PEO-DA/0.5%CNF/1%GelMA, (**c**) 12%PEO-PPO-PEO-DA/0.5%CNF/3%GelMA, and (**d**) 12%PEO-PPO-PEO-DA/0.5%CNF/5%GelMA injectable hydrogels.

**Figure 10:**
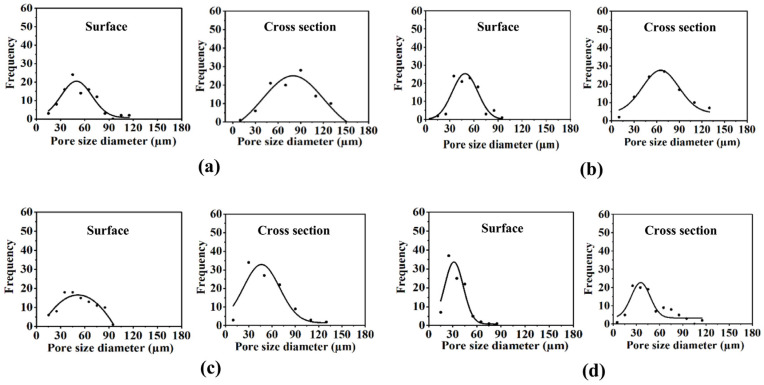
Pore size diameter distribution at the top surface and in the cross-section of (**a**) 12%PEO-PPO-PEO-DA/0.5%CNF, (**b**) 12%PEO-PPO-PEO-DA/0.5%CNF/1%GelMA, (**c**) 12%PEO-PPO-PEO-DA/0.5%CNF/3%GelMA, and (**d**) 12%PEO-PPO-PEO-DA/0.5%CNF/5%GelMA injectable hydrogels.

The FE-SEM micrographs of the topical surface and cross-section of the PEO-PPO-PEO-DA/CNF and all the PEO-PPO-PEO-DA/CNF/GelMA show highly microporous structures with an interconnecting network (Figure 9) with a pore size diameter of surface range of 11–128 µm and a cross-section range of 8–140 µm (Figure 10 and Table 5). All the hydrogels show high porosity of 75–99%. With the increasing GelMA concentration in the hydrogels, porosity increases and the pore size diameter range of the surface and cross-section of the hydrogels show a narrower range (Table 5). Especially, all the hydrogels show suitable pore sizes and porosity that meet the scaffold requirements (pore sizes ranging from 7–350 µm and porosity of ≥55%).

##### Gel Fraction, Water Uptake, and Swelling

The PEO-PPO-PEO-DA/CNF and PEO-PPO-PEO-DA/CNF/GelMA injectable hydrogels meet the property requirements with high gel fraction (82–83%), water uptake (86–88%), water swelling (622–730%), and porosity (74–98%) (Table 6). Interestingly, water uptake and swelling of the hydrogels are augmented with GelMA concentration.

##### Mechanical Properties

**Figure 11:**
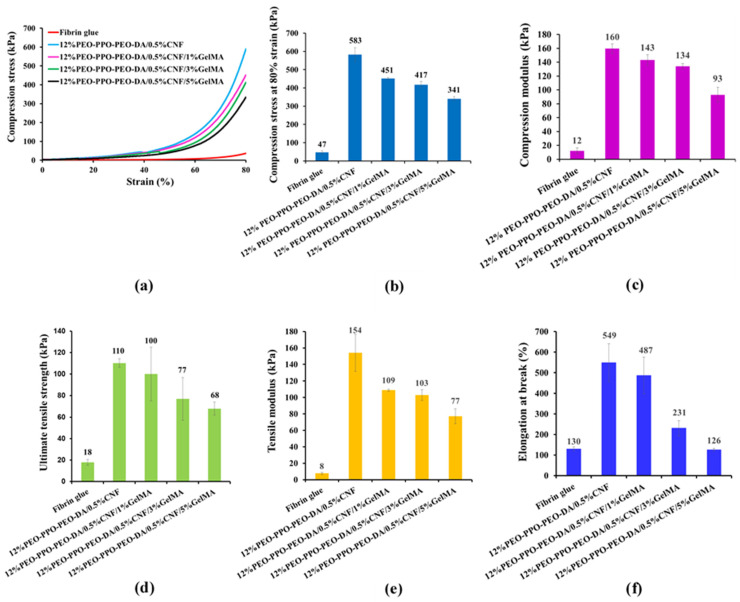
Mechanical properties, (**a**) Compression stress-strain curve, (**b**) Compression stress at 80% strain, (**c**) Compression modulus, (**d**) Tensile strength, (**e**) Tensile modulus, and (**f**) Elongation at break of PEO-PPO-PEO-DA/CNF, PEO-PPO-PEO-DA/CNF/GelMA injectable hydrogels and fibrin glue.

The mechanical properties of the injectable hydrogels and fibrin glue are shown in Figure 11 and Table 7. All the injectable hydrogels show remarkably higher mechanical properties than those of the fibrin glue (Figure 10). Unfortunately, the mechanical properties of the injectable hydrogels decreased with the increasing GelMA from 1 to 5% (*w*/*v*) resulting in PPO-PEO-DA/0.5%CNF/5%GelMA showing lower mechanical properties than hydrogel scaffold requirements (Table 7). However, the CNF-reinforced PEO-PPO-PEO-DA with the addition of GelMA from 1 to 3% show good mechanical properties, such as compression strength (417–451 kPa), compression modulus (134–143 kPa), ultimate tensile strength (77–100 kPa), tensile modulus (103–109 kPa), and elongation at break (231–487%), which meet the hydrogel scaffold requirements (Table 7).

##### Chemical Interaction

**Figure 12:**
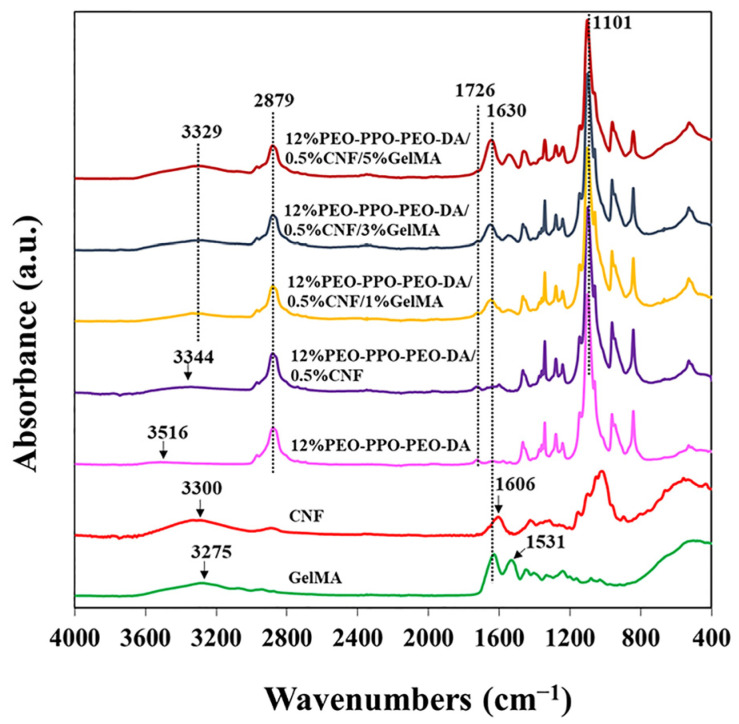
FTIR spectra of CNF, GelMA, and injectable hydrogels.

FTIR spectra of CNF, GelMA, and hydrogels are shown in Figure 12. CNF shows a characteristic absorption band at 3600–3000 cm^−1^ (OH stretching) and 1606 cm^−1^ (O-H bending vibrations owing to absorbed water with some contributions from carboxylate groups). PEO-PPO-PEO-DA exhibits a typical absorption band at 3600–3400 cm^−1^ (OH stretching), 2879 cm^−1^ (C–O–C stretching vibration of PEO), and 1726 cm^−1^ (C=O stretching vibration of ester groups and 1101 cm^−1^ (symmetrical stretching vibration –CH_3_ of PPO). GelMA demonstrates a significant absorption band at 3600–3000 cm^−1^ (OH stretching), 1630 cm^−1^ (C=O stretching vibration), and 1531 cm^−1^ (N-H stretching vibration). Characteristic peaks for CNF, PEO-PPO-PEO-DA, and GelMA can be observed in the PEO-PPO-PEO-DA/CNF/GelMA injectable hydrogel which confirms the presence of all the components in the hydrogel. The absorbance at 1630 and 3329 cm^−1^ augments with the increasing amounts of GelMA in the PEO-PPO-PEO-DA/CNF/GelMA injectable hydrogel. Interestingly, some characteristic peaks of the CNF, PEO-PPO-PEO-DA, and GelMA are shifted in the composite injectable hydrogel indicating interaction occurring among the different components of the hydrogels. The FTIR spectra of the PEO-PPO-PEO-DA/CNF reveal the OH stretching peak of CNF shifting from 3300 to 3344 cm^−1^, probably because of the hydrogen bonds between –OH of PEO-PPO-PEO-DA and –OH of CNF. The FTIR spectra of PEO-PPO-PEO-DA/CNF/GelMA reveal the OH stretching peak of GelMA shifting from 3275 to 3329 cm^−1,^ hinting hydrogen bonds between –OH of GelMA and –OH of PEO-PPO-PEO-DA or –OH of CNF, N-H stretching vibration of GelMA shifting from 1531 to 1549 cm^−1^ suggesting H-bonding between C=O of GelMA with –OH of CNF. The FTIR spectra of PEO-PPO-PEO-DA/CNF and PEO-PPO-PEO-DA/CNF/GelMA injectable hydrogels suggest intermolecular H-bonding between PEO-PPO-PEO-DA and CNF, between PEO-PPO-PEO-DA and GelMA and between CNF and GelMA indicating good compatibility of composite hydrogels.

#### 3.3.2. In Vitro Cell Culture Studies

##### Cell Cytotoxicity

**Figure 13:**
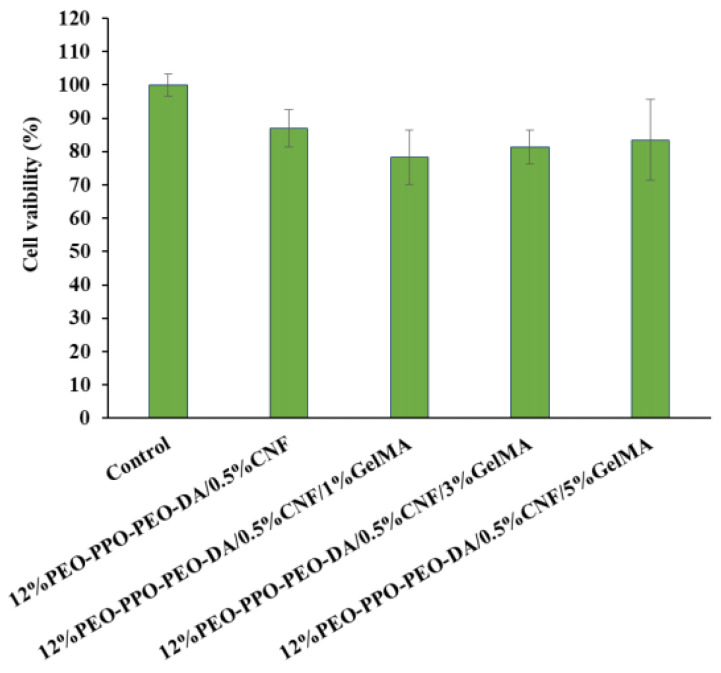
Cell viability of CSPCs after incubation in the extracted medium of injectable hydrogels for 24 h.

The cytotoxicity test aims at assessing the ability of certain chemicals to destroy living cells. Cytotoxicity measurements are important indicators for evaluating toxicological endpoints of biomedical materials because of their ease of performance, high speed, and high sensitivity, and they can reduce animal experimentation. From the in vitro cytotoxicity of PEO-PPO-PEO-DA/CNF and PEO-PPO-PEO-DA/CNF/GelMA, injectable hydrogels were assessed using an MTT assay with CSPCs. The cell viabilities of CSPCs after incubation in all extracted medium of hydrogels for 24 h were approximately 78–87% (Figure 13), indicating that PEO-PPO-PEO-DA/CNF and PEO-PPO-PEO-DA/CNF/GelMA injectable hydrogel are non-cytotoxic as confirmed by cell viabilities > 70%.

##### Cell Proliferation

**Figure 14:**
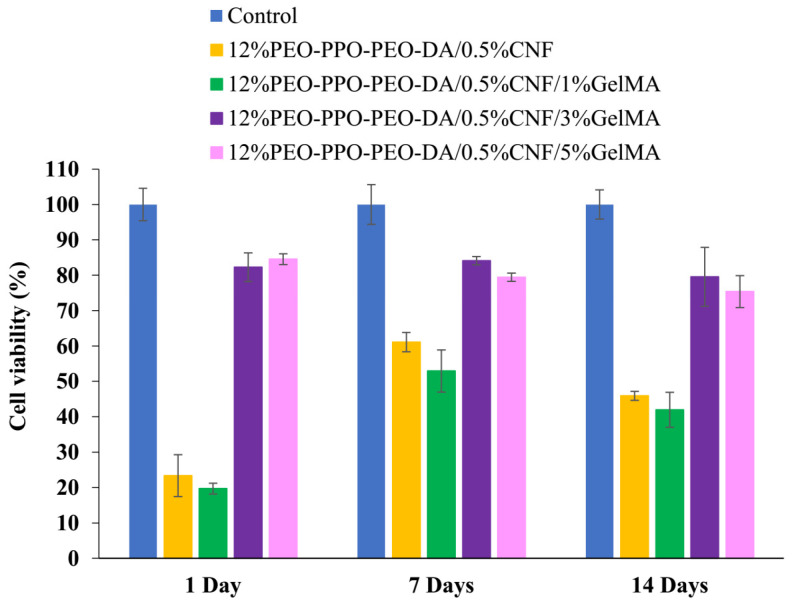
Cell viability of CSPCs after culture in injectable hydrogels for 1, 7, and 14 days.

**Figure 15:**
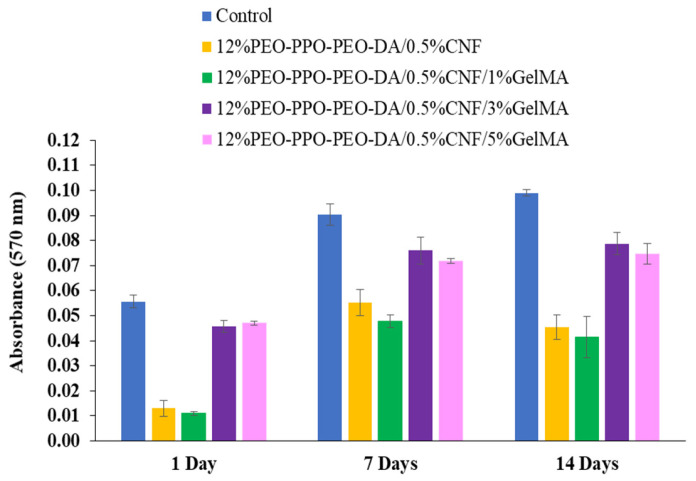
Absorbance as a function of cell proliferation during 14 days of in vitro culture.

Scaffolds must display cell adhesion properties that promote the growth of seed cells for enhancement of cell proliferation. The proliferation of CSPCs cultured on PEO-PPO-PEO-DA/CNF with various concentrations of GelMA for up to 1, 7, and 14 days was carried out by MTT assay. The different CSPCs proliferation in various concentrations of GelMA were compared by cell viability as shown in Figure 14. Absorbance as a function of cell proliferation for understanding the overall trend of cell activity is shown in Figure 15. Cell viability in 12%PEO-PPO-PEO-DA/0.5%CNF and 12%PEO-PPO-PEO-DA/0.5%CNF/1%GelMA hydrogels was not significantly different (*p* > 0.05) at every time point. On Day 1, 12%PEO-PPO-PEO-DA/0.5%CNF and 12%PEO-PPO-PEO-DA/0.5%CNF/1%GelMA hydrogels showed low cell viability of 23 and 20%, respectively, On Day 7, cell viability in 12%PEO-PPO-PEO-DA/0.5%CNF and 12%PEO-PPO-PEO-DA/0.5%CNF/1%GelMA hydrogel increased from 23 to 61 and 20 to 53%, respectively. On Day 14, cell viability of 12%PEO-PPO-PEO-DA/0.5%CNF and 12%PEO-PPO-PEO-DA/0.5%CNF/1%GelMA hydrogels slightly decreased from 61 to 46 and 53 to 42%, respectively. Although these hydrogels are non-cytotoxic, they gave low cell viability. This suggests that these hydrogels have low cell adhesion and proliferation in the long term. On the other hand, PEO-PPO-PEO-DA/CNF hydrogel incorporating 3% and 5% (*w*/*v*) of GelMA, showed high cell viability of more than 80% at every time point implying good cell adhesion and proliferation and non-toxicity. Especially, 12%PEO-PPO-PEO-DA/0.5%CNF/3%GelMA and 12%PEO-PPO-PEO-DA/0.5%CNF/5%GelMA hydrogels showed significantly higher cell viability (*p* < 0.05) than 12%PEO-PPO-PEO-DA/0.5%CNF and 12%PEO-PPO-PEO-DA/0.5%CNF/1%GelMA hydrogels at every time point. The results confirm our hypothesis that the incorporation of GelMA at optimum concentration into an injectable hydrogel can promote cell adhesion and proliferation.

All of the experiment results verify that CNF-reinforced PEO-PPO-PEO-DA/GelMA injectable hydrogels represent a system alternative to existing therapeutics. This system is important to develop a novel scaffold for reducing the surgical wound area and healing time by minimally invasive surgery instead of open incisions. These hydrogels enhance the physicochemical properties which ensure superior performance compared to conventional hydrogels, fibrin glues, and other developed hydrogel materials (summarized in Table 3). Especially, this injectable hydrogel system significantly promotes cell proliferation and also shows good physicochemical properties which meet meniscus tissue engineering requirements. The results indicate that the mechanical characteristics of the injectable hydrogels and cell viability are significantly improved via incorporating CNF and GelMA, respectively, into the injectable hydrogels which proves our hypothesis correct.

## 4. Conclusions

We have shown here CNF-PEO-PPO-PEO-DA/GelMA injectable hydrogel after curing with UV light within 10 min with a thickness of 2 mm, CNF significantly improves its mechanical properties. The incorporation of GelMA into the injectable hydrogel has led to promote cell proliferation of human cartilage stem/progenitor cells. At optimum formulation, 12%PEO-PPO-PEO-DA/0.5%CNF/3%GelMA injectable hydrogels significantly promote cell proliferation (cell viability > 80%) and also show good physicochemical properties which meet tissue engineering requirements. To sum up, our data show that the novel injectable hydrogel is a good candidate for applications in meniscus tissue engineering.

## Figures and Tables

**Table 1:** The crystallinity index (CrI) (%) of CP, BCP, and CNF.

Sample	Amorphous Region Peak Intensity (a.u.)	Crystalline Region Peak Intensity (a.u.)	CrI (%)
CP	5441	6725	19.10
BCP	5070	9550	46.91
CNF	3820	6663	42.67

**Table 2:** Pore size diameter and porosity of injectable hydrogel.

Formulations	Pore Size Diameter (Surface) (µm)	Pore Size Diameter (Cross-Section) (µm)	Porosity (%)
12%PEO-PPO-PEO-DA	14–128	12–141	93.11 ± 3.14
12%PEO-PPO-PEO-DA/0.3%CNF	19–128	14–168	79.54 ± 3.13
12%PEO-PPO-PEO-DA/ 0.5%CNF	12–118	16–140	74.48 ± 1.74
12%PEO-PPO-PEO-DA/ 0.7%CNF	11–112	9–81	64.92 ± 4.00

**Table 3:** The comparison of different orthopedic tissue engineering materials in mechanical properties.

Materials	Compression Strength (kPa)	Compression Modulus (kPa)	Tensile Strength (kPa)	Tensile Modulus (kPa)	Elongation at Break (%)	References
Stearyl methacrylate (C18M)/silk fibroin hydrogel	17.10	/	/	/	/	[23]
Nano-hydroxyapatite/ poly(L-glutamic acid)-dextran hydrogel	51.00	/	/	/	/	[24]
Hydroxyapatite coated a chitosan-polyvinyl alcohol hydrogel	66.90	109.90 ± 7.00	/	/	/	[25]
Peptide loaded oxidized dextran/GelMA hydrogel	110.00	136.50 ± 11.60	/	/	/	[26]
Genipin cross-linked gelatin hydrogel	70.00	300.00 ± 30.00	16.00	36.59 ± 3.98	46.00	[27]
Fibrin glue	46.71 ± 8.87	11.98 ± 4.45	17.99 ± 2.68	7.69 ± 1.30	129.28 ± 8.78	This work
12%PEO-PPO-PEO-DA hydrogel	398.01 ± 26.82	120.86 ± 9.47	57.32 ± 4.25	96.68 ± 9.61	450.64 ± 37.40	
12%PEO-PPO-PEO-DA/ 0.3%CNF hydrogel	428.64 ± 30.49	142.87 ± 2.48	69.60 ± 17.24	139.47 ± 16.49	499.34 ± 53.95	
12%PEO-PPO-PEO-DA/ 0.5%CNF hydrogel	582.55 ± 38.18	159.54 ± 7.06	110.24 ± 4.00	154.10 ± 22.60	548.97 ± 92.06	
12%PEO-PPO-PEO-DA/ 0.7%CNF hydrogel	518.42 ± 56.27	150.02 ± 15.70	78.44 ± 5.42	148.28 ± 4.39	566.07 ± 16.40	
Hydrogel scaffold requirement	≥100	≥100	≥50	≥100	≥100	[28]

Note: / means not mentioned.

**Table 4:** Gel fraction, water uptake, and water swelling of injectable hydrogel.

Formulations	Gel Fraction (%)	Water Uptake (%)	Water Swelling (%)
12%PEO-PPO-PEO-DA	83.72 ± 0.38	86.53 ± 0.03	642.59 ± 1.60
12%PEO-PPO-PEO-DA/0.3%CNF	82.85 ± 0.78	86.18 ± 0.30	623.80 ± 16.00
12%PEO-PPO-PEO-DA/ 0.5%CNF	82.59 ± 1.00	86.16 ± 0.14	622.80 ± 7.24
12%PEO-PPO-PEO-DA/ 0.7%CNF	81.30 ± 0.89	86.07 ± 0.22	617.97 ± 11.33

**Table 5:** Pore size diameter and porosity of injectable hydrogel.

Formulations	Pore Size Diameter (Surface) (µm)	Pore Size Diameter (Cross-Section) (µm)	Porosity (%)
12%PEO-PPO-PEO-DA/ 0.5%CNF	12–118	16–140	74.48 ± 1.74
12%PEO-PPO-PEO-DA/ 0.5%CNF/1%GelMA	11–97	16–138	92.60 ± 0.87
12%PEO-PPO-PEO-DA/ 0.5%CNF/3%GelMA	13–92	9–126	95.62 ± 0.87
12%PEO-PPO-PEO-DA/ 0.5%CNF/7%GelMA	12–83	8–114	98.63 ± 0.88

**Table 6:** Gel fraction, water uptake, and water swelling of the injectable hydrogels.

Formulations	Gel Fraction (%)	Water Uptake (%)	Water Swelling (%)
12%PEO-PPO-PEO-DA/ 0.5%CNF	82.59 ± 1.00	86.16 ± 0.14	622.80 ± 07.24
12%PEO-PPO-PEO-DA/ 0.5%CNF/1%GelMA	82.39 ± 0.51	86.19 ± 0.51	624.77 ± 27.38
12%PEO-PPO-PEO-DA/ 0.5%CNF/3%GelMA	82.29 ± 0.28	87.14 ± 1.03	680.62 ± 61.03
12%PEO-PPO-PEO-DA/ 0.5%CNF/7%GelMA	82.05 ± 0.62	87.95 ± 0.23	730.31 ± 16.00

**Table 7:** The comparison of injectable hydrogel and fibrin glue in mechanical properties.

Materials	Compression Strength (kPa)	Compression Modulus (kPa)	Tensile Strength (kPa)	Tensile Modulus (kPa)	Elongation at Break (%)	References
Fibrin glue	46.71 ± 8.87	11.98 ± 4.45	17.99 ± 2.68	7.69 ± 1.30	129.28 ± 8.78	This work
12% F127DA/0.5%CNF	582.55 ± 38.18	159.54 ± 7.06	110.24 ± 4.00	154.10 ± 2.60	548.97 ± 92.06	
12% F127DA/0.5%CNF/ 1%GelMA	451.33 ± 6.03	143.25 ± 7.60	100.00 ± 25.00	108.79 ± 1.37	487.00 ± 88.71	
12% F127DA/0.5%CNF/ 3%GelMA	417.33 ± 17.90	133.96 ± 4.18	77.00 ± 20.00	102.87 ± 6.62	231 ± 36.45	
12% F127DA/0.5%CNF/ 5%GelMA	340.67 ± 11.59	92.94 ± 10.75	68.00 ± 6.00	77.18 ± 8.96	126 ± 6.56	
Hydrogel scaffold requirements	≥ 100	≥ 100	≥ 50	≥ 100	≥ 100	[28]

## Data Availability

Not applicable.
